# Conjugated Linoleic Acid Production in Pine Nut Oil: A *Lactiplantibacillus plantarum Lp-01* Fermentation Approach

**DOI:** 10.3390/foods13162472

**Published:** 2024-08-06

**Authors:** Gang Wei, Ge Wu, Jiajia Sun, Yi Qi, Qi Zhao, Fengde Xu, Zhi Zhang, Lanzhi Peng

**Affiliations:** 1 School of Ocean and Tropical Medicine, Guangdong Medical University, Zhanjiang 524023, China; wgang@gdmu.edu.cn (G.W.); mizuyama_masaki@163.com (G.W.); qiyi7272@gdmu.edu.cn (Y.Q.); zhaoqi17@mails.ucas.ac.cn (Q.Z.); 13827198525@163.com (F.X.); 2 The Marine Biomedical Research Institute of Guangdong Zhanjiang, Zhanjiang 524023, China; 3 College of Forestry, Northeast Forestry University, 26 Hexing Road, Xiangfang District, Harbin 150040, China; 2022020083@nefu.edu.cn; 4 College of Life Sciences, Northeast Forestry University, 26 Hexing Road, Xiangfang District, Harbin 150040, China

**Keywords:** conjugated linoleic acid, *Lactiplantibacillus plantarum*, pine nut oil, microbial synthesis

## Abstract

Conjugated linoleic acid (CLA) is a class of bioactive fatty acids that exhibit various physiological activities such as anti-cancer, anti-atherosclerosis, and lipid-lowering. It is an essential fatty acid that cannot be synthesized by the human body and must be derived from dietary sources. The natural sources of CLA are limited, predominantly relying on chemical and enzymatic syntheses methods. Microbial biosynthesis represents an environmentally benign approach for CLA production. Pine nut oil, containing 40–60% linoleic acid, serves as a promising substrate for CLA enrichment. In the present study, we developed a novel method for the production of CLA from pine nut oil using *Lactiplantibacillus plantarum* (*L. plantarum*) *Lp-01*, which harbors a linoleic acid isomerase. The optimal fermentation parameters for CLA production were determined using a combination of single-factor and response surface methodologies: an inoculum size of 2%, a fermentation temperature of 36 °C, a fermentation time of 20 h, and a pine nut oil concentration of 11%. Under these optimized conditions, the resultant CLA yield was 33.47 μg/mL. Gas chromatography analysis revealed that the fermentation process yielded a mixture of c9, t11CLA and t10, c12 CLA isomers, representing 4.91% and 4.86% of the total fatty acid content, respectively.

## 1. Introduction

Conjugated linoleic acid (CLA) is a specific class of linoleic acid, which encompasses all stereo and positional isomers [1]. CLA is one of the essential fatty acids for the human body and must be obtained from dietary sources. Among these isomers, c9, t11-CLA and t10, c12-CLA are the most physiologically active. Research indicates that CLA possesses biologically functions, including improving bone density, enhancing immune function, promoting lipid oxidation and decomposition, and exhibiting potential protective effects against cancer, osteoporosis, and atherosclerosis, as well as aiding in weight management and lipid reduction [2,3].

Natural CLA is primarily produced by microbial metabolism in the digestive tract of ruminants, and where it accumulates in the meat and dairy products of ruminants [4]. In addition, CLA is also found in some seafood, vegetable oils, and fish oils. Nevertheless, CLA is present in only minor amounts in such foods, ranging from 0.6 mg/g to 9.4 mg/g. This is far below the recommended daily intake of 3.2 g of CLA for the human body, and cannot be achieved by consuming natural foods alone [5]. Thus, increasing the dietary intake of CLA at a lower cost is of significant importance.

At present, the common methods to increase the content of CLA in food are enzymatic, chemical, and microbial syntheses. Microbial biosynthesis is the most environmentally friendly method for CLA synthesis [6]. *Lactic acid bacteria* (LAB) strains are among the most studied in microbial synthesis and possess genes that produce linoleic acid isomerase [7,8,9,10]. *Lactiplantibacillus plantarum* (*L. plantarum*) is a strain of *Lactobacillus* with high CLA production capacity [11]. The isomers cis-9, trans-11 (c9, t11-CLA) and trans-10, cis-12 (t10, c12-CLA) are two configurations of conjugated linoleic acid with high physiological activity [4,12,13,14]. The content of conjugated linoleic acid in these biologically active isomers is consistently high in the CLA products produced by *L. plantarum*, resembling the CLA isomers found in natural foods.

Research on plant-derived CLA indicates that no physiologically active isomers have been identified in natural vegetable oils, thus making it improbable to obtain CLA in large quantities from natural plants. Consequently, substantial quantities of CLA can be synthesized by chemical and biological methods, using linoleic acid (LA) or LA-rich vegetable oils as substrates [15]. Common vegetable oils for this purpose include castor [16], sunflower [17], safflower [18], and flaxseed oils [19]. Pine nut oil, derived from the seeds of pine trees, contains 90% unsaturated fatty acids, including linolenic, linoleic, oleic acids, and the unique pinolenic acid, with linoleic acid comprising 60% of the unsaturated fatty acids [20,21]. LA is known to lower blood lipids, prevent atherosclerosis, and cardiovascular diseases [22,23,24]. CLA possesses a multitude of physiological benefits, including anti-cancer, anti-oxidation, and anti-atherosclerosis properties, as well as enhancing immunity, bone density, preventing diabetes, and promoting growth. Pine nuts are a nutritious food and a traditional Chinese medicine. Owing to their unique flavor and health benefits, the demand for pine nuts and pine nut oil has been growing in the global market. The fat content of red pine seed oil has reached 70%, with linoleic acid being the predominant fatty acid, accounting for 47.6% of the total fatty acid content, making it a suitable source for the production of CLA [25].

In our previous study, we isolated a strain of *L. plantarum Lp-01* from sauerkraut. It was confirmed by gene sequencing that *L. plantarum Lp-01* harbors the gene for linoleic acid isomerase. The aim of the present study was to investigate the process of converting LA from pine nut oil to CLA by *L. plantarum Lp-01*. We sought to determine the optimum time, temperature, initial bacterial inoculum size, and the quantity of pine nut oil used for CLA production by *L. plantarum Lp-01*. The results of this study will provide a theoretical foundation for the application of *L. plantarum Lp-01* and the development of pine nut oil functional food enriched in CLA.

## 2. Materials and Methods

### 2.1. Material and Chemicals

*L. plantarum Lp-01* is a strain that we have isolated in our laboratory. The strain has been identified to possessing a linoleic acid isomerase gene. The CLA standard mixture (*cis*-9, *trans*-11, and *trans*-10, *cis*-12) was acquired from Sigma Aldrich (St. Louis, MO, USA). Linoleic acid, with a purity of 95%, was acquired from Shanghai Yuanye Biotechnology Co., Ltd. (Shanghai, China). Korean pine seeds were obtained from Yichun city (Heilongjiang province, China). The mechanical crushing extraction method was utilized to obtain pine nut oil using a vacuum oil press machine. The vacuum oil press machine was purchased from Zhengzhou Zhengke Machinery Manufacturing Co., Ltd., Zhengzhou, China.

### 2.2. Identification of Linoleic Acid Isomerase (LAI) Gene of L. plantarum Lp-01

According to the gene sequences of linoleate isomerase in *L. plantarum* retrieved from GenBank (accession numbers: HQ831447.1, FR732045.1, KU555936.1), a homologous coding box was identified using Vector NTI Advance 10 software analysis. Primers were designed using Primer Premier 5 software (Version 5, Palo Alto, CA, USA) based on the nucleotide sequence of the identified coding box (Table 1) and synthesized by Shanghai Shenggong Biological Engineering Co., Ltd. (Shanghai, China).

*L. plantarum Lp-01* was inoculated into 5 mL of liquid MRS medium at 37 °C for 10 h, followed by centrifugation at 10,000 rpm for 10 min at 4 °C to pellet the bacterial cells. Genomic DNA of *L. plantarum Lp-01* was extracted following the protocol of the Ezup column bacterial genomic DNA extraction kit. The PCR mixture, 25 µL in volume, contained dNTPs (10 mM) 0.2 µL, distilled water 10.1 µL, primers F and R (10 mM each) 0.5 µL, Taq polymerase (5 U/µL) 0.2 µL, template cDNA 1 µL, and 2x × GC Buffer I 12.5 µL. PCR amplification was performed under the following conditions: initial denaturation at 95 °C for 5 min, followed by 10 cycles of 94 °C for 30 s, 63 °C for 30 s with a decrease of 0.5 °C per cycle, then 30 cycles of 95 °C for 30 s, 58 °C for 30 s, and 72 °C for 30 s, with a final extension at 72 °C for 10 min. PCR products of the target gene from *L. plantarum Lp-01* were analyzed by 1% agarose gel electrophoresis, visualized and documented using a gel imaging system, and the bands of interest were excised and recovered. The LAI PCR products of *L. plantarum Lp-01* were sequenced by Shanghai Shenggong Bioengineering Co., Ltd. (Shanghai, China).

### 2.3. Microorganism Culture and Substrate 

The strain was incubated in MRS solid medium (pH 6.5) for stationary culture at 37 °C for 24 h, and then passed three times according to the inoculum of 1% to serve as the activated experimental strain. Pine nut oil (450 mg) was mixed with Tween 80 (1 mL), followed by the addition of water to achieve a final volume of 100 mL. An ice bath was used form the pine nut oil emulsion through ultrasonication with a TL-1000Y sonicator (Jiangsu Tianxiang Instrument Co., Ltd., Yancheng, China) at approximately 800 W, applying 5-s pulses with 5-s intervals for a total of 25 min. Pine nut oil emulsion (4.5 mg/mL) was filtered and sterilized using a microporous filter, with a pore diameter of the microporous filter membrane of 0.22 μm (Millex, Shanghai Weixi Biotechnology Co., Ltd., Shanghai, China).

### 2.4. Optimization of Fermentation Conditions for CLA Production

The fermentation culture conditions, such as time, temperature, pine nut oil content, and inoculum size, were optimized by assessing the respective parameters. MRS medium was sterilized at 121 °C for 20 min, to which the pine nut oil concentration and activated strain were added for fermentation culture. The effects of fermentation temperature, pine nut oil content (2% to 16%), and inoculum size (0.5% to 6%) on CLA production were investigated. The culture medium was cultured with oscillation at various temperatures (20 °C to 48 °C) for 24 h. For the effect of fermentation time on CLA production, the fermentation mixture contained 2% activated strain and 10% pine nut oil concentration in the MRS medium (100 mL) and was cultured with oscillation at 37 °C. After 10 h of incubation, samples were collected every 5 h to measure the CLA content.

Response surface methodology (RSM) was utilized to optimize the fermentation process of CLA from *L. plantarum Lp-01*. Based on the Box–Behnken design principle, inoculum size, fermentation temperature, fermentation time, and pine nut oil content were chosen as independent variables, and CLA yield was designated as the response variable for the experimental design with four factors (A, inoculum size; B, fermentation temperature; C, fermentation time; D, pine nut oil concentration) at three levels, as depicted in Table 2.

### 2.5. CLA Extraction and Determination 

The fermentation supernatant was collected by centrifugation (6000× *g*, 10 min) (2-16R, Hunan Henuo Instrument Equipment Co., Ltd., Changsha, China) at 4 °C. The supernatant was mixed with a hexane (1:6, *v*/*v*) solution, and the hexane extract was washed with distilled water. Then, the organic phase was dehydrated with anhydrous sodium sulfate and evaporated using a rotary evaporator at 30 °C. UV spectrum analysis methods were used to determine the content of CLA. The standard sample of CLA was scanned from 200 nm to 300 nm, and the characteristic absorption peak of CLA was identified at 233 nm. The CLA standard curve (*y* = 0.11216*x* + 0.0549, R^2^ = 0.9896) was constructed for calculating the concentration of CLA in each sample. The hexane extract of the MRS broth lacking any strain was used as a blank control. The formation of fermentation products in the fermentation liquid with and without substrates was monitored at 233 nm using a UV/Vis spectrophotometer (UV-5500PC, Shanghai Precision Instrument Co., Ltd., Shanghai, China) [26].

### 2.6. Gas Chromatography–Mass Spectrometry (GC-MS) Analysis 

The GC-MS analysis of fermentation products was carried out by a Trace1310ISQ (Thermo, Waltham, MA, USA) system with an HP-88 capillary column (100 m × 0.25 mm × 0.2 µm, Santa Clara, CA, USA). Initially, the column temperature was 100 °C for 1 min, followed by an increase to 200 °C at a rate of 10 °C/min, and then increased to 250 °C at a rate of 5 °C/min, and finally increased to 270 °C at a rate of 2 °C/min for 3 min. Helium was used as the carrier gas with a flow rate of 1.2 mL/min. Ion source and interface temperature were set to 280 °C. The mass spectra were obtained by electron ionization at 70 eV with a scanning range of 30~400 *m*/*z* at a rate of 0.44 scans/s.

### 2.7. Bioinformatics Analysis

Initially, we submitted the *L. plantarum Lp-01* LAI PCR amplification product to Shanghai Bio-Engineering Co., Ltd. (Shanghai, China) for sequencing, which yielded a linoleic acid isomerase gene of 1308 base pairs in length. Utilizing the BLAST program from the NCBI database (https://blast.ncbi.nlm.nih.gov/Blast.cgi, accessed on 27 June 2024), we performed a similarity search against the existing linoleic acid isomerase genes within the database. Subsequently, we constructed a phylogenetic tree using MEGA11 software (version 11). We predicted transmembrane segments using the TMpred Server, modeled the tertiary structure of the protein with SWISS-MODEL (http://swissmodel.expasy.org/), and visualized it using Swiss-PDB Viewer based on homology modeling [12].

### 2.8. Statistical Analysis

All the experiments were conducted in triplicate, and the data are presented as the mean value ± standard deviation. The figures were generated using Origin Software Version 9.1 (Origin Lab Corp., Northampton, MA, USA), and statistical analysis was performed using ANOVA tests (*p* < 0.05) with SPSS, version 11.0 (SPSS Inc., Chicago, IL, USA). Design-Expert^®^ software, version 10.0.7 (Stat-Ease, Inc., Minneapolis, MN, USA), was utilized for quadratic multiple regression analysis of the experimental results, with *p* < 0.05 indicating statistical significance. 

## 3. Results and Discussion

### 3.1. Identification, Analysis, and Phylogenetic Analysis of LAI in L. plantarum Lp-01

**Figure 1:**
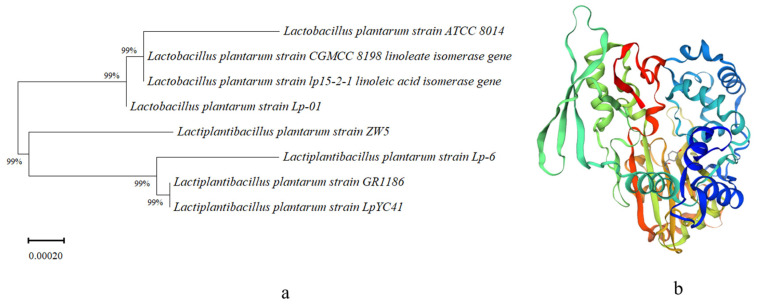
Bioinformatic analysis of LAI in *L. plantarum Lp-01*. (**a**) Phylogenetic tree analysis of LAI in *L. plantarum Lp-01*. (**b**) Tertiary structure prediction of *L. plantarum Lp-01* LAI by the SWISS-MODEL based on homology modeling.

As shown in Figure 1, the vector sequence was excluded from the determination results, revealing the *L. plantarum Lp-01* LAI gene to be 1308 base pairs in length. The NCBI BLAST program was used to compare the sequence with others of linoleate isomerase in the NCBI databases, accessed via the NCBI BLAST interface. The homology between the *Lp-01* LAI gene (PP955898) and other linoleate isomerase genes from different strains was clearly demonstrated, with 99% sequence identity observed in comparison to the following: *L. plantarum* lp15-2-1 (HQ831447.1), *L. plantarum* ATCC 8014 (FR732045.1), and *L. plantarum* CGMCC 8198 (KU555936.1). The gene sequence has been deposited in GenBank with the accession number PP955898. Homology analysis of *L. plantarum Lp-01* was conducted using TreeView software available on the NCBI website, as shown in Figure 1a.

**Figure 2:**
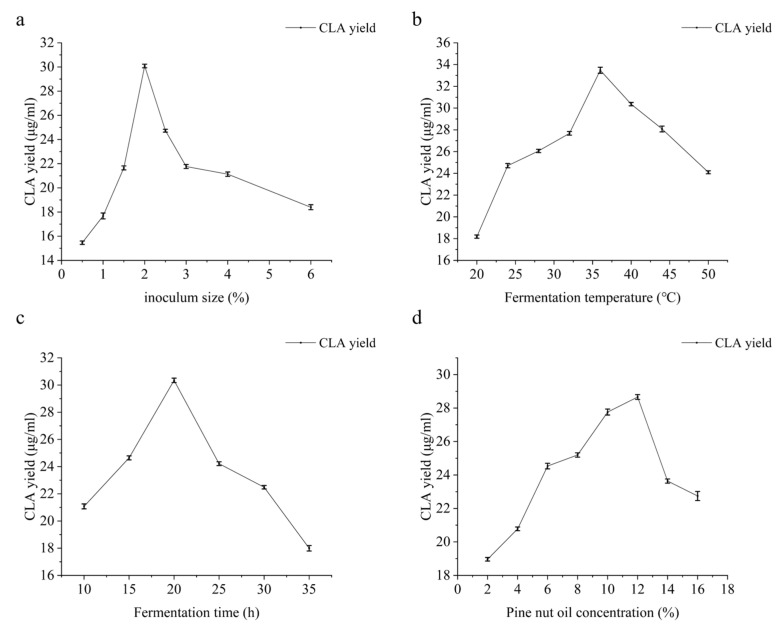
Effects of different fermentation parameters on CLA yield. (**a**) Effect of inoculum size on CLA yield. (**b**) Effect of fermentation temperature on CLA yield. (**c**) Effect of fermentation time on CLA yield. (**d**) Effect of pine nut oil concentration on CLA yield.

The full-length cDNA of *L. plantarum Lp-01* LAI consists of 1308 base pairs, and the amino acid sequence was inferred through codon translation from the linoleate isomerase gene sequence. The predicted open reading frame (ORF) of the cDNA encodes a protein consisting of 431 amino acids, with a molecular weight of 49.54 kDa and a theoretical isoelectric point (pI) of 5.14. Furthermore, the tertiary structure of this protein was established using SWISS-MODEL and visualized with Swiss-PDB Viewer based on homology modeling, as shown in Figure 2b. LAI catalyzes the conversion of linoleic acid to biologically active CLA. Predicting its tertiary structure allows researchers to gain a deeper understanding of the enzyme’s active site and how substrates bind and undergo catalytic reactions.

### 3.2. Effect of Inoculum Size on CLA Yield

The effect of inoculum size (8 × 10^10^ CFU/mL) on CLA yield is shown in Figure 2a. The CLA yield ranged from 0.5% to 6%, peaking at 2% of the inoculum size. The CLA yield increased rapidly with inoculum sizes from 0.5% to 2%, after which it showed a decreasing trend upon exceeding 2%. The conversion of LA to CLA is primarily catalyzed by the enzyme linoleic acid isomerase. An increase in the inoculum can elevate enzyme content, enhancing CLA production. However, if the inoculum size is too large, the growth of the strain will be affected. In addition, a decrease in nutrients in the medium will inhibit the metabolism of the strain, leading to a reduced enzyme activity of linoleic acid isomerase. Consequently, the maximum CLA yield of 30.08 µg/mL was achieved at a 2% inoculum size.

### 3.3. Effect of Fermentation Temperature on CLA Yield

Fermentation temperature is an important factor affecting the activity of linoleic acid isomerase and the conversion rate. As shown in Figure 2b, when the temperature ranged from 20 °C to 36 °C, the CLA yield increased with the temperature. However, beyond this range, the CLA yield decreased. Lower temperatures cannot supply enough heat for the fermentation reaction, which is detrimental to bacterial growth and enzyme production. Excessive temperature accelerates the denaturation process of the linoleic acid isomerase enzyme, resulting in its inactivation.

### 3.4. Effect of Fermentation Time on CLA Yield

The effect of fermentation time on CLA yield is shown in Figure 2c. Short fermentation times resulted in lower CLA yields and enzyme activities. As shown in the figure, the CLA yield initially increased but later decreased as fermentation time extended. When the fermentation time was fixed at 20 h, the CLA yield peaked at 30.34 µg/mL. The absence of further improvement in CLA yield with extended fermentation time might be due to product inhibition. As fermentation time increased, a reduction in inducers and an accumulation of reaction products inhibited CLA transformation, leading to decreased CLA yields. Additionally, the accumulation of excess acids in the environment can also disrupt the bacteria’s enzyme-producing metabolism.

### 3.5. Effect of Pine Nut Oil Concentration on CLA Yield

Specific microbial strains with linoleic acid isomerase activity are inoculated into the medium, enabling the growth cells to ferment LA or utilize LA-containing substrates concurrently with their reproduction, and carry out biotransformation to synthesize CLA [27]. As shown in Figure 2d, the CLA yield gradually increased with the concentration of pine nut oil emulsion and subsequently decreased with the increase in substrate concentration. When the concentration of pine nut oil was set to 12%, the CLA yield reached a maximum of 28.66 µg/mL. LAI is an enzyme whose synthesis can be induced, and its synthesis is affected by the substrate concentration. Pine nut oil emulsion, as a substrate, can induce the synthesis of LAI but may also inhibit the growth of Lactobacillus. This suggests that the high concentration of pine nut oil inhibits bacterial growth, resulting in a reduced CLA yield.

Pine nut oil emulsion as a substrate can induce the synthesis of linoleic acid isomerase, yet it may also inhibit the growth of *lactic acid bacteria*. This may be attributed to the high concentration of pine nut oil emulsion inhibiting bacterial growth, thereby leading to a decrease in CLA yield. Rainio et al. [28] observed that the conversion of linoleic acid initially added during the *Propionibacterium* fermentation process for CLA production was higher than that added mid-way. The conversion of LA to CLA essentially mitigates its toxic effects on the microbial cells; hence, an appropriate substrate concentration is essential for enhancing CLA yield.

### 3.6. Response Surface Method to Optimize the Fermentation Process of L. plantarum Lp-01

The potential of *L. plantarum Lp-01* isolated from sauerkraut to produce conjugated linoleic acid by fermenting pine nut oil has been investigated. We examined the effects of various microbial culture conditions, such as temperature, time, and substrate concentration, on the production of conjugated linoleic acid [9]. The response surface methodology was applied for optimizing the fermentation process to maximize CLA yield. 

#### 3.6.1. Predicted Model and Analysis of Variance

The process by which *L. plantarum Lp-01* produces CLA from Korean pine seed oil was optimized using RSM. The results of 29 experimental runs by employing the Box–Behnken design and including the predicted CLA yields from the design are presented in Table 3. The CLA yield was analyzed using multiple regression techniques. The results of the complete second-order model and ANOVA are given in Table 3. The Design-Expert^®^ software, version 10.0.7, was to perform the quadratic multiple regression analysis on the experimental data, yielding the following regression model:

*Y* = 33.84 + 1.22 × A − 0.35 × B + 1.63 × C − 0.9833 × D − 0.7 × A × B − 2.20 × A × C − 0.4 × A × D − 0.45 × B × C − 0.4B × D− 1.15 × C × D − 3.47 × A^2^ − 1.89 × B^2^ − 3.74 × C^2^ − 4.32 × D^2^

The ANOVA results, which evaluate the predictive factors and the model, are presented in Table 4. *F*-values and *p* values are utilized to assess the significance of each coefficient. The greater the F-values and the lower the *p* values, the more significant the coefficient. The factor contribution rate, obtained from the F test, is as follows: C > A > D > B. The effect of the inoculum size on CLA yield was very significant (*p* < 0.01), and the effect of culture time on CLA yield was also very significant (*p* < 0.01). The interaction between factors A and C was highly significant (*p* < 0.01). Furthermore, the model has an R^2^ of 0.9721 and an adjusted R^2^ (R^2^Adj) of 0.9442, indicating that the predicted values fit well with the experimental values, thereby validating the rationality of the four-factor design.

#### 3.6.2. Optimization and Validation for Fermentation Conditions

**Figure 3:**
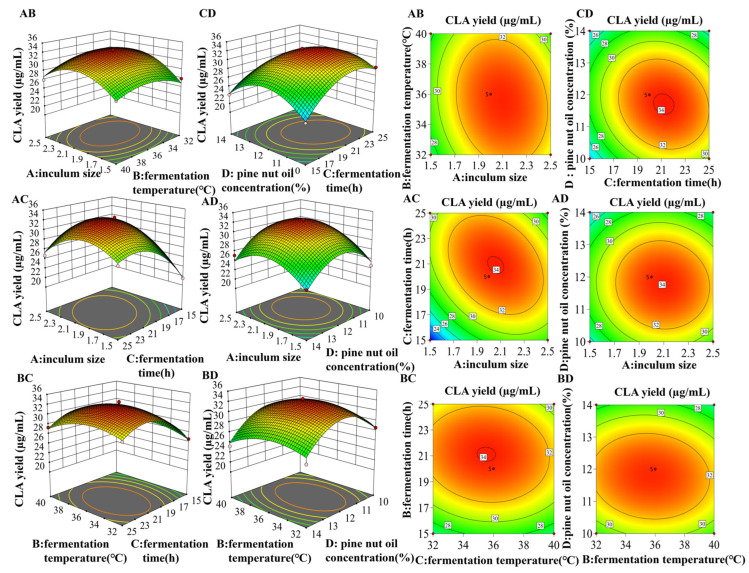
Response surface and contour plots utilizing the Box–Behnken design for optimizing CLA yield: (**AB**) inoculum size vs. fermentation temperature; (**AC**) inoculum size vs. fermentation time; (**AD**) inoculum size vs. pine nut oil concentration; (**BC**) fermentation temperature vs. fermentation time; (**BD**) fermentation temperature vs. pine nut oil concentration; (**CD**) fermentation time vs. pine nut oil concentration. A: Inoculum size, B: fermentation temperature, C: fermentation time, D: pine nut oil concentration.

The contour lines and 3D response surface generated from the model were used to determine the influence of various experimental factors on CLA yields and are depicted in Figure 3. The contour map reveals the significance of interactions between the factors. An oval contour line implies a significant interaction between two factors, whereas a circular contour line indicates no significant interaction. Utilizing the Design-Expert^®^ software version 10.0.7, the optimal fermentation conditions for CLA production were established as an inoculum size of 2.07%, a fermentation temperature of 35.62 °C, a fermentation time of 20.12 h, and a pine nut oil concentration of 11.7%. Additional experiments, conducted thrice under optimum fermentation conditions, were taken into account to consider practical operation and equipment parameters. The CLA yield was 33.47 μg/mL, closely aligning with the predicted value of 33.75 μg/mL, with a discrepancy of 0.8%. The response surface methodology (RSM) model’s predictive accuracy, as evidenced by the close agreement between the experimental and predicted values, underscores the model’s robustness. The high R^2^-value and the lack of significant difference between the model’s predicted and actual outcomes confirm the model’s reliability for optimizing the fermentation conditions.

Our findings indicate that pH, temperature, and fermentation time are critical factors affecting the bioconversion of linoleic acid to CLA by *lactic acid bacteria*. The optimized conditions determined using the RSM model were consistent with previous studies highlighting the sensitivity of microbial fermentation to these parameters. However, it is essential to recognize that, in addition to the RSM’s success in optimizing CLA production in this study, other factors such as metal ion, oxygen levels, and microbial strain variability, could also influence the fermentation process [29,30]. Future research should consider multifactorial optimization, including these additional variables [31].

### 3.7. The Fermentation Products’ Composition Analysis 

#### 3.7.1. Results of the Extraction Liquid of Fermentation Product Scanned by Ultraviolet Wavelength

**Figure 4:**
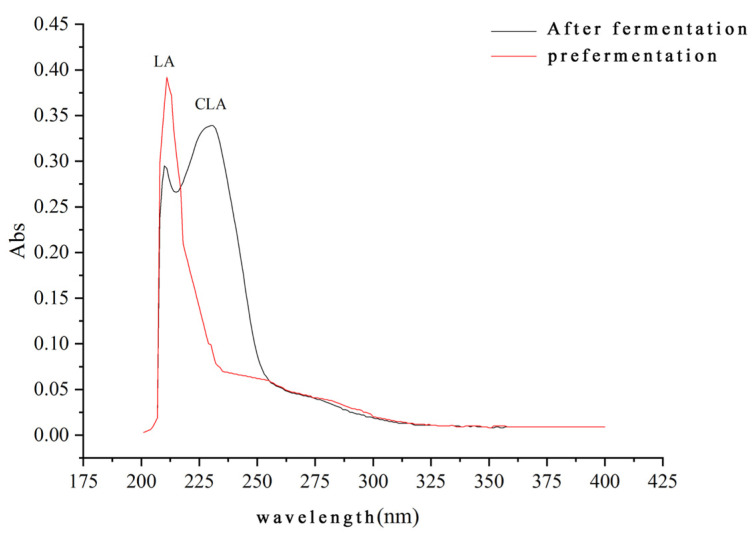
Spectral analysis of fermentation extract.

A discernible accumulation of CLA was detected in the medium after 20 h of fermentation using active growing cells. The maximum absorption values indicate a range of 200 nm to 205 nm for LA and 233 nm to 234 nm for CLA. Figure 4 shows that post transformation, the light absorption value of the extraction solution at 205 nm decreased while at 233 nm increased, indicating that a portion of LA in the pine nut oil was transformed to CLA by *L. plantarum Lp-01*.

#### 3.7.2. Gas Chromatography (GC) Figure of Fatty Acid Methyl Ester from Fermentation

**Figure 5:**
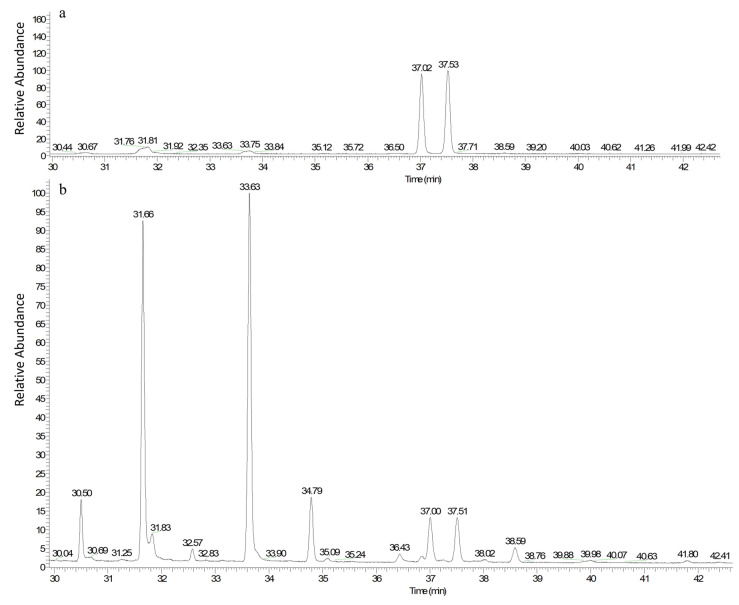
Analysis of pine nut oil fermentation products. (**a**) Gas chromatography chromatogram of conjugated linoleic acid standard samples. (**b**) Gas chromatography chromatogram of pine nut oil fermentation products.

The individual chromatograms of the CLA mixture standard and fermentation broth, obtained by GC, are displayed in Figure 5a,b. In Figure 5a, the peak time of *c*-9 and *t*-11 CLA was 30.02 min, and that of *t*10, *c*12-CLA was 37.53 min. Figure 5b shows the GC figure of the fatty acid methyl ester from the fermentation product of pine nut oil emulsion fermented by *L. plantarum Lp-01*. Compared to the GC chromatogram of the standard CLA mixture in Figure 5a, the peak time of *c*9, *t*11-CLA in Figure 5a was 37.02 min, and its proportion in the fermentation broth product was 4.91%. In Figure 5a, the peak time of t10, c12-CLA was 37.52 min, and its proportion in the fatty acid methyl ester product of the fermentation broth was 4.86%. Regarding retention times, other peaks in Figure 5b are not associated with CLA.

## 4. Conclusions

This study successfully designed, optimized, and simulated a method for extracting conjugated linoleic acid (CLA) from pine nut oil through fermentation with *L. plantarum Lp-01*. Utilizing a Box–Behnken design (BBD) and response surface methodology (RSM), optimal conditions for inoculum size, fermentation temperature, time, and pine nut oil concentration (2%, 36 °C, 20 h, and 11%, respectively, for both response variables) were simultaneously combined to achieve the highest extraction yield of CLA. Under these optimal conditions, the experimental yield of CLA was 33.47 μg/mL, which is in good agreement with the predicted value of 0.08%. 

The proportions of c-9, t-11 CLA and t-10, c-12 CLA in the product fatty acid methyl ester were 4.91% and 4.86%, respectively, and the overall CLA content was 9.77%. Direct fermentation for the production of conjugated linoleic acid (CLA) via microbial fermentation is associated with several limitations, such as high costs, difficult-to-manage control conditions, and low yields, impeding its industrial-scale application. The enzymatic catalysis yields singular CLA active isomers under conditions that are more easily controlled and result in higher product yields. To enhance CLA production, further mutagenesis of *L. plantarum Lp-01* is necessary, facilitating the purification of linoleic acid isomerase from the fermentation products. Subsequently, the utilization of linoleic acid isomerase can lead to the production of pine nut oil enriched with a higher concentration of CLA.

In conclusion, the biotransformation of pine nut oil to produce CLA using *L. plantarum Lp-01* constitutes a promising strategy for the enrichment of pine nut oil with this beneficial compound. The optimization of fermentation conditions has led to a significant increase in CLA yield, offering a potential alternative to chemical synthesis. However, further research is required to address the scalability, economic viability, and impact on oil quality prior to its adoption for commercial applications.

## Figures and Tables

**Table 1:** Primer design.

ID	Primer Sequence	
	Forward	Reverse
1	AATAAGAATCATCCGATGGCTAA	GTTCCGTGAAGATCATCTGGTAT
2	AATAAGAATCATCCGATGGCTAA	GTTCCGTGAAGATCATCTGGTAT
3	GAATAAGAATCATCCGATGGCTA	GTTCCGTGAAGATCATCTGGTAT

**Table 2:** Experimental factors’ level code.

Level	Factors			
	A (%)	B (°C)	C (h)	D (%)
−1	1.5	32	15	10
0	2	36	20	12
1	2.5	40	25	14

**Table 3:** Box–Behnken design and results for the CLA yield.

Run Numbers	A: Inoculum Size/%	B: Temperature/ °C	C: Time/ h	D: Pine Nut Oil Concentration/%	CLA Yield/ μg/mL
1	2.00	32.00	20.00	10.00	28.6
2	2.50	40.00	20.00	12.00	27.9
3	2.00	32.00	15.00	12.00	26.6
4	1.50	36.00	15.00	12.00	21.5
5	2.50	36.00	20.00	10.00	28.7
6	2.00	36.00	20.00	12.00	31.3
7	2.00	36.00	20.00	12.00	34.4
8	2.00	36.00	15.00	14.00	24.1
9	1.50	36.00	20.00	14.00	24.6
10	2.00	40.00	15.00	12.00	27.3
11	2.00	36.00	20.00	12.00	34.3
12	2.00	32.00	20.00	14.00	26.2
13	2.00	32.00	15.00	10.00	23.7
14	1.50	36.00	20.00	10.00	24.6
15	2.50	32.00	20.00	12.00	31.0
16	2.00	36.00	20.00	12.00	33.6
17	1.50	36.00	25.00	12.00	28.8
18	1.50	40.00	20.00	12.00	27.6
19	2.50	36.00	15.00	12.00	28.2
20	2.00	36.00	20.00	12.00	33.3
21	2.00	40.00	20.00	14.00	25.2
22	2.00	36.00	25.00	10.00	30.0
23	2.00	36.00	25.00	14.00	25.8
24	2.50	36.00	25.00	12.00	26.7
25	2.00	32.00	25.00	12.00	30.4
26	2.00	40.00	25.00	12.00	29.3
27	1.50	32.00	20.00	12.00	27.9
28	2.00	40.00	20.00	10.00	29.2
29	2.50	36.00	20.00	14.00	27.1

**Table 4:** Analysis of variance for the fitted regression model.

Variables	Sum of Squares	df	Mean Square	*F*-Value	*p*-Value	
Model	303.96	14	21.71	34.85	<0.0001	significance
A	17.76	1	17.76	28.51	0.0001	
B	1.47	1	1.47	2.36	0.1468	
C	32.01	1	32.01	51.39	<0.0001	
D	11.60	1	11.60	18.62	0.0007	
AB	1.96	1	1.96	3.15	00979	
AC	19.36	1	19.36	31.08	<0.0001	
AD	0.6400	1	0.6400	1.03	0.3280	
BC	0.8100	1	0.8100	1.30	0.2733	
BD	0.6400	1	0.6400	1.03	0.3280	
CD	5.29	1	5.29	8.49	0.0113	
A2	78.10	1	78.10	125.37	<0.0001	
B2	23.29	1	23.29	37.39	<0.0001	
C2	90.97	1	90.97	146.02	<0.0001	
D2	121.05	1	121.05	194.31	<0.0001	
Residual	8.72	14	0.6230			
Lack of fit	7.79	10	0.7790	3.34	0.1280	Not significance
Pure error	0.9320	4	0.2330			
Cor.Total	312.68	28				

## Data Availability

The data that support the findings of this study are available from the corresponding author, ZP, upon reasonable request.
